# Precision measurement of cardiac structure and function in cardiovascular magnetic resonance using machine learning

**DOI:** 10.1186/s12968-022-00846-4

**Published:** 2022-03-10

**Authors:** Rhodri H. Davies, João B. Augusto, Anish Bhuva, Hui Xue, Thomas A. Treibel, Yang Ye, Rebecca K. Hughes, Wenjia Bai, Clement Lau, Hunain Shiwani, Marianna Fontana, Rebecca Kozor, Anna Herrey, Luis R. Lopes, Viviana Maestrini, Stefania Rosmini, Steffen E. Petersen, Peter Kellman, Daniel Rueckert, John P. Greenwood, Gabriella Captur, Charlotte Manisty, Erik Schelbert, James C. Moon

**Affiliations:** 1 Institute of Cardiovascular Science, University College London, London, UK; 2 Bart’s Heart Centre, St Bartholomew’s Hospital, West Smithfield, London, EC1A 7BE UK; 3 MRC Unit for Lifelong Health and Ageing, University College London, London, UK; 4 National Heart, Lung, and Blood Institute, National Institutes of Health, Bethesda, USA; 5 Data Science Institute, Imperial College London, London, UK; 6 William Harvey Research Institute, Queen Mary University of London, London, UK; 7 National Amyloidosis Centre, University College London, London, UK; 8 Sydney Medical School, University of Sydney, Sydney, Australia; 9 Department of Clinical, Internal, Anesthesiology and Cardiovascular Sciences, Sapienza University of Rome, Rome, Italy; 10 Biomedical Image Analysis Group, Department of Computing, Imperial College London, London, UK; 11 Leeds Institute of Cardiovascular and Metabolic Medicine, University of Leeds, Leeds, UK; 12 Department of Medicine, University of Pittsburgh School of Medicine, Pittsburgh, USA; 13 Minneapolis Heart Institute East, Saint Paul, MN USA

**Keywords:** Machine learning, Cardiovascular imaging, Cardiac magnetic resonance, Ventricular function, Image processing

## Abstract

**Background:**

Measurement of cardiac structure and function from images (e.g. volumes, mass and derived parameters such as left ventricular (LV) ejection fraction [LVEF]) guides care for millions. This is best assessed using cardiovascular magnetic resonance (CMR), but image analysis is currently performed by individual clinicians, which introduces error. We sought to develop a machine learning algorithm for volumetric analysis of CMR images with demonstrably better precision than human analysis.

**Methods:**

A fully automated machine learning algorithm was trained on 1923 scans (10 scanner models, 13 institutions, 9 clinical conditions, 60,000 contours) and used to segment the LV blood volume and myocardium. Performance was quantified by measuring precision on an independent multi-site validation dataset with multiple pathologies with n = 109 patients, scanned twice. This dataset was augmented with a further 1277 patients scanned as part of routine clinical care to allow qualitative assessment of generalization ability by identifying mis-segmentations. Machine learning algorithm (‘machine’) performance was compared to three clinicians (‘human’) and a commercial tool (cvi42, Circle Cardiovascular Imaging).

**Findings:**

Machine analysis was quicker (20 s per patient) than human (13 min). Overall machine mis-segmentation rate was 1 in 479 images for the combined dataset, occurring mostly in rare pathologies not encountered in training. Without correcting these mis-segmentations, machine analysis had superior precision to three clinicians (e.g. scan-rescan coefficients of variation of human vs machine: LVEF 6.0% vs 4.2%, LV mass 4.8% vs. 3.6%; both *P* < 0.05), translating to a 46% reduction in required trial sample size using an LVEF endpoint.

**Conclusion:**

We present a fully automated algorithm for measuring LV structure and global systolic function that betters human performance for speed and precision.

**Supplementary Information:**

The online version contains supplementary material available at 10.1186/s12968-022-00846-4.

## Background

Measures of cardiac size, mass, and function derived from imaging are some of the most fundamental biomarkers in medicine. For example, left ventricular (LV) ejection fraction (LVEF) determines selection for drug therapy in heart failure [1–3], detects myocardial injury (e.g. in cardio-oncology) [4], acts as a gatekeeper for ~ $9 billion spent per year on cardiac devices, and acts as surrogate endpoints for drug development and outcome prediction [5–7].

LVEF was initially proposed as a simple way of measuring heart function using cardiac catheterization [8]. The introduction of imaging modalities such as echocardiography, cardiac computed tomography and cardiovascular magnetic resonance (CMR) permitted absolute blood and myocardial volume measurements. CMR is accepted as the best technique for measuring cardiac structure and global systolic function (i.e., LVEF) [9]. Image acquisition is standardized and can be delivered in as little as 15 min [10, 11], but the image analysis process can take as long, requiring analysis by a clinician, which imparts variability because of intra- and inter-operator differences [12, 13].

Recent developments in deep learning using convolutional neural networks (CNN)—computational models inspired by the architecture of the human brain—have revolutionized automated image analysis [14]. The potential of CNNs in healthcare is being recognized; for example, a deep learning system has been shown to improve on human performance for detecting breast cancer in mammograms [15]. Many CNN applications in cardiac image segmentation have been described and deployed within commercial packages [16–18], but none surpass human performance and most algorithms are not directly compared to human analysis on the same data, nor validated on independent clinical datasets [19].

We hypothesized that a carefully trained, fully automated machine learning analysis could be developed and proven to exceed human performance on any CMR scanner and any (non-congenital) disease. This approach requires a means of evaluating and comparing CMR measures of myocardial structure and function, but this is hampered by the lack of a truth standard. Most existing approaches report measurement accuracy, treating expert analysis (or a consensus thereof) as a truth standard, but this is fundamentally flawed because of the inherent variability and subjectivity of humans. We therefore concentrate on measurement precision (reproducibility) and develop an evaluation framework using an independent dataset to quantify measurement precision (reproducibility), which determines clinical smallest detectable interval change and research study sample size.

## Methods

**Fig. 1:**
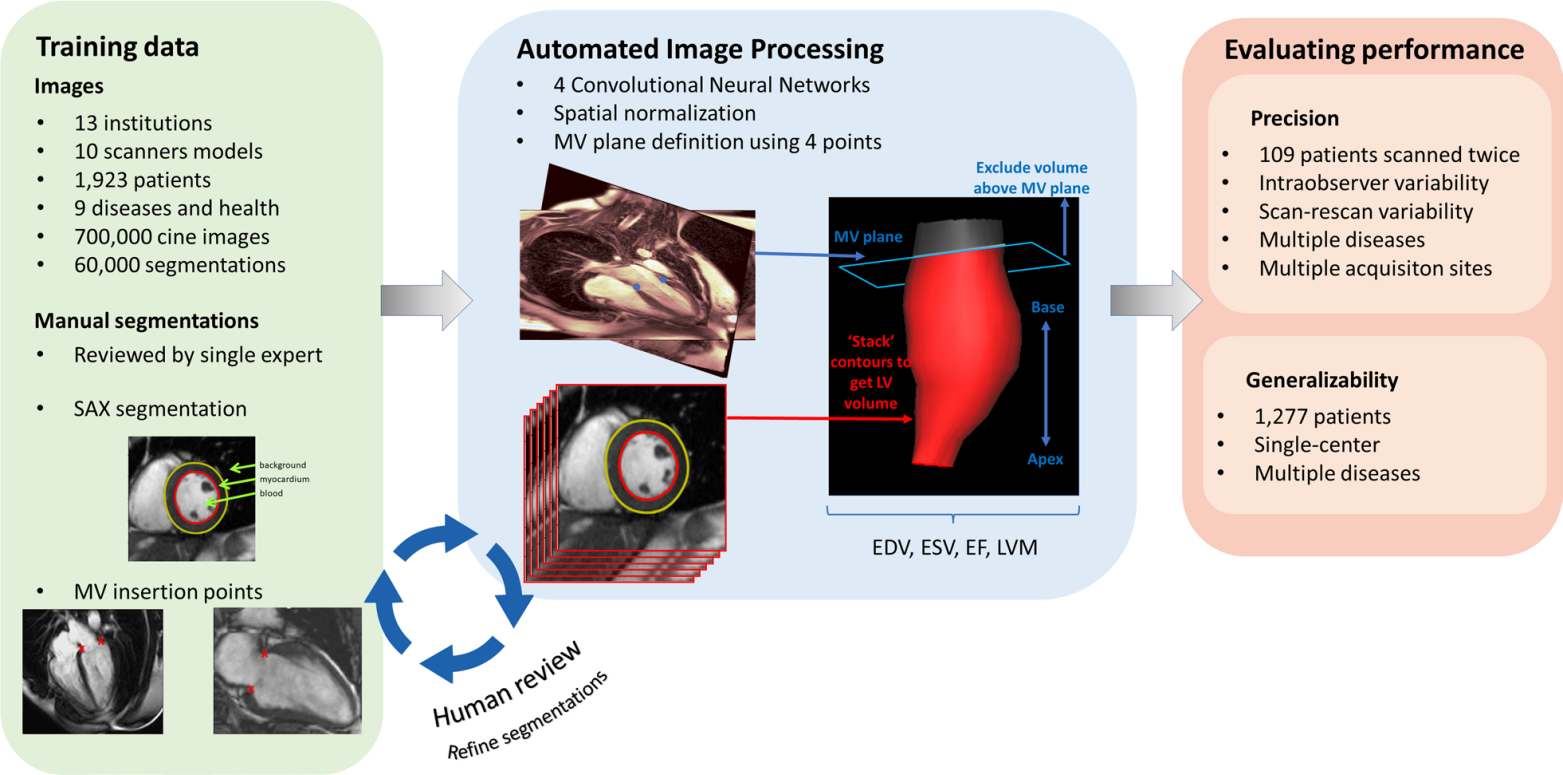
Overview of study design. A training set of segmented images from 1932 patients with multiple diseases from multiple centres were used to train four convolutional neural networks (CNNs). CNN segmentations were combined to measure left ventricular (LV) cavity volumes, systolic function and myocardial mass. Machine segmentations were compared to clinical segmentations on an independent dataset to measure precision. *EDV* end diastolic volume, *ESV* end systolic volume, *EF* ejection fraction, *LVM* LV mass, *MV* mitral valve, *SAx* short axis

An overview of the study design is illustrated in Fig. 1 and detailed below.

### Automated image analysis approach

We use deep fully CNNs to annotate (“segment”) the LV blood pool and myocardium from CMR image datasets [20, 21]. Here we describe the CNN architecture, the data on which it was trained, and three additional steps to improve model performance: spatial normalization, mitral annular plane correction, and iterative training segmentation refinement.

All image acquisition reflected standard international recommendations [11] and conventional CMR cine images were acquired of two-chamber (2C), four-chamber (4C) views and a stack of short axis (SAx) slices.

#### Convolutional neural networks

Four CNNs were used in total: a SAx model (containing the blood pool only), a 2-chamber (2Ch) model and a 4-chamber (4Ch) model. Diastole was identified as the cardiac phase with largest blood volume and a further CNN, trained only in diastole, was used to segment the myocardium.

**Fig. 2:**
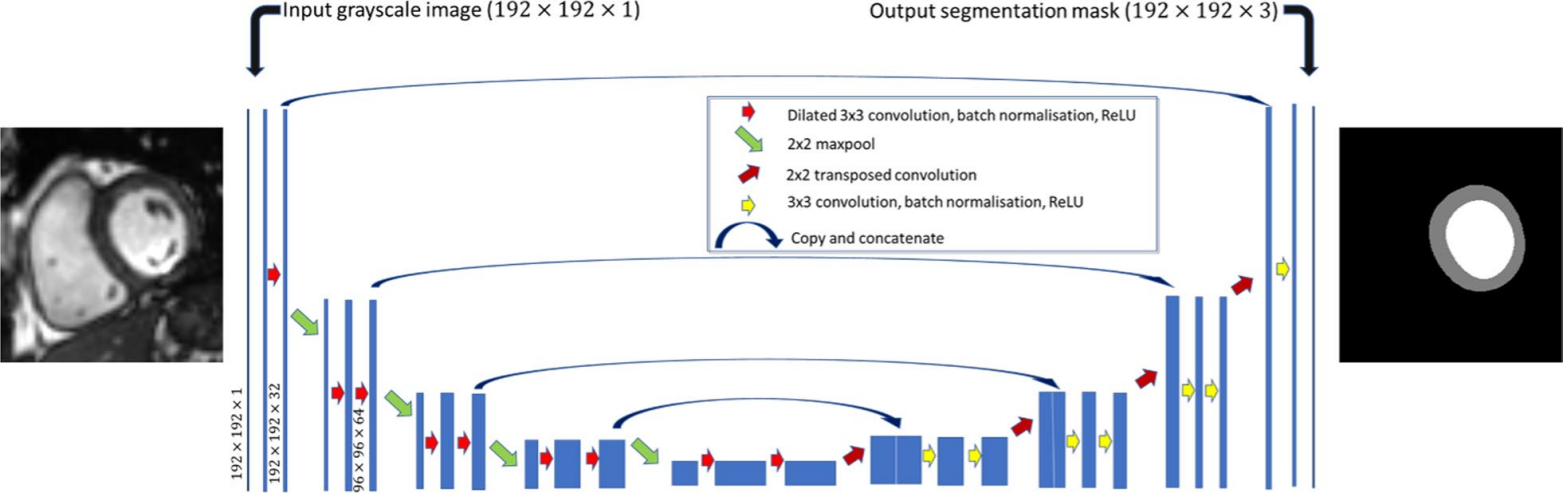
Structure of the Unet used for short axis image segmentation. The model takes a grayscale CMR image with dimension 192 × 192 and creates a segmentation mask of the same dimension with 3 channels (one channel for each of: LV blood pool (white), myocardium (gray) and background (black)). The Unet used for long axis segmentations were the same, but image sizes and final layer were different—see Additional file 1: Table S1 for full details

A U-net architecture [21] was adopted with dilatated (Atrous) convolutions and batch normalization [22]—a schematic of the network is shown in Fig. 2. The Adam algorithm was used to optimize each model using a learning rate of 10^–4^ with no decay and image augmentation (scaling, rotation, translation) was used to artificially increase the variability in the training set—the technical details of each model is listed in Additional file 1: Table S1.

#### Spatial normalisation

**Fig. 3:**
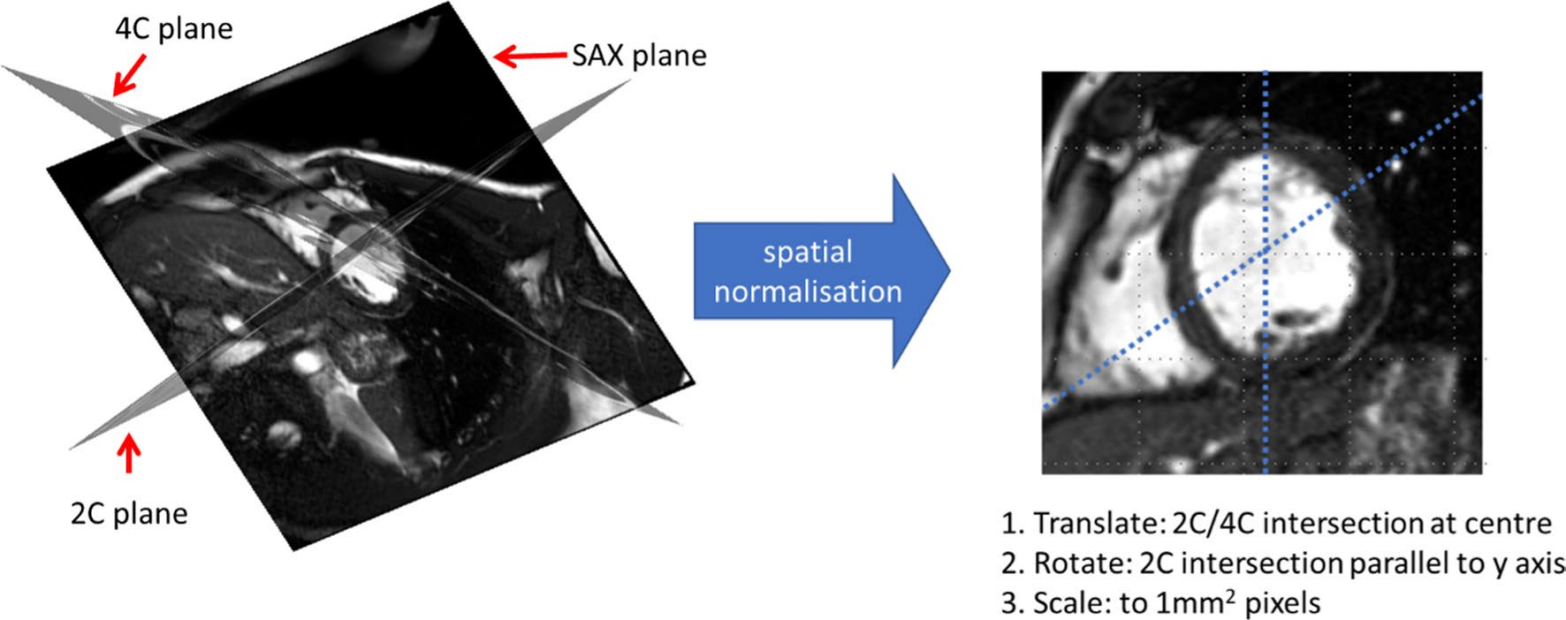
Spatial normalisation. The geometric relationship between the SAx, 2Ch and 4Ch planes are known—the three planes are overlaid in 3D in the left image. Spatial normalisation of each image is performed by transformation to a normalised reference frame as shown in the right image. *2Ch* 2-chamber, *4Ch* 4-chamber, *SAx* short-axis

All images were normalized to a standard reference frame by: centering the SAx images to the point of intersection with the long axis (Lax) images (2Ch and 4Ch), rotating so the SAx and 2Ch intersection aligned to the y-axis (meaning the LV outflow tract was always orientated in the same way), and scaling to an in-plane pixel size of 1 mm^2^—see Fig. 3.

#### Defining the base of the myocardium

**Fig. 4:**
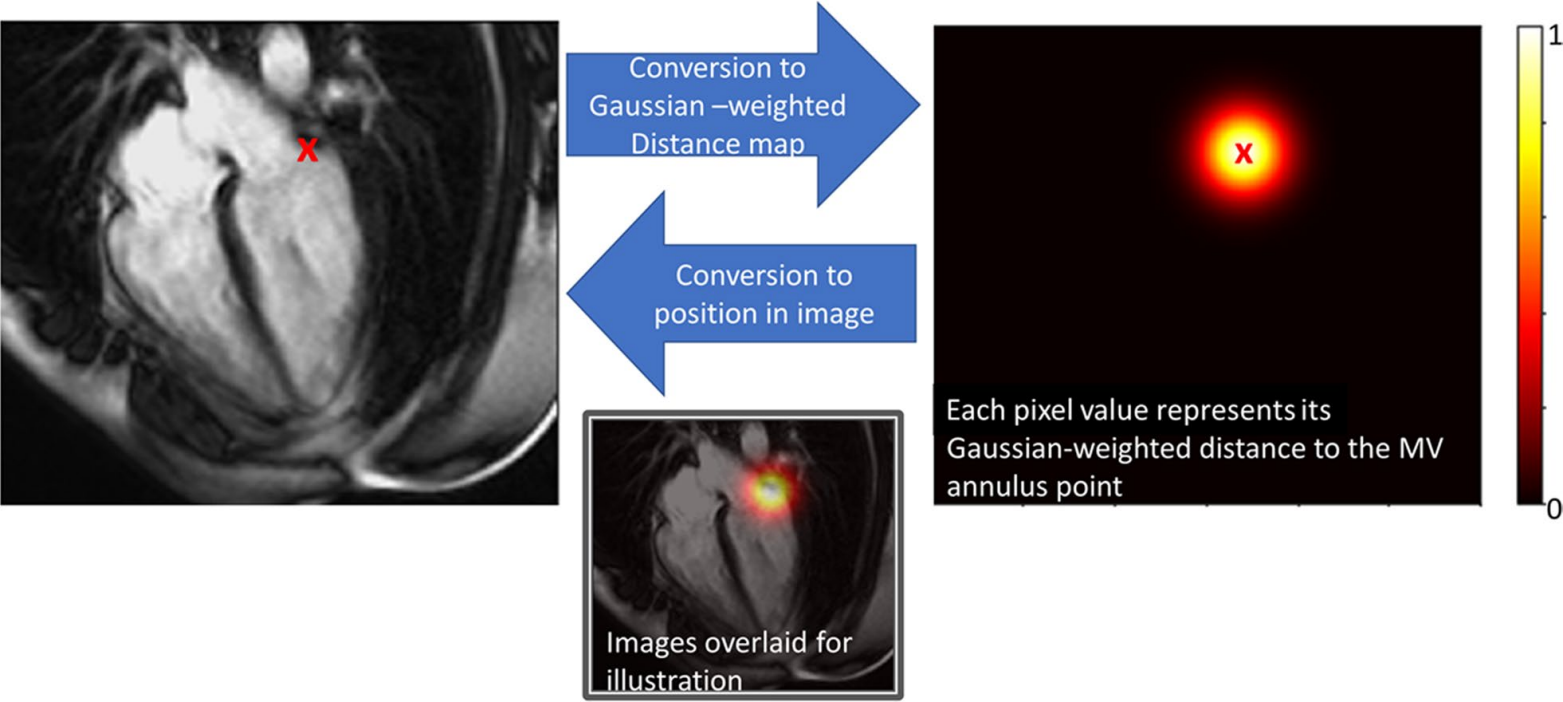
Mitral annular position encoding. The image on the left shows the lateral mitral annular point overlaid on the CMR image. The image on the right was created by measuring the distance to the mitral annular point from each pixel position and weighting with a Gaussian function; the position of the point is overload for illustration. The bottom image shows the CMR image and distance map overlaid. For clarity, only one of the two points is shown here. *MV* mitral valve

Since the basal extent of the myocardium is difficult to determine from SAx images alone, we used the 2Ch and 4Ch images to define the mitral annulus, below which any volume was discarded [23]. A plane was fitted to the mitral annulus by a least-squares fit to two points on each of the 2Ch and 4Ch at the intersection of the mitral annulus and myocardium. The geometric transformation needed to map each image slice into a common 3-dimensional space can be calculated from information in the digital imaging and communications in medicine (DICOM) header and the volume formed by stacking the SAx contours can be trimmed by the mitral valve plane, discarding any volume below it (Fig. 1). While the point coordinates could be predicted directly by adding an additional fully connected layer to our CNN, we achieved more robust results using a U-net to predict a Gaussian weighted distance map, where each pixel represents the distance to the point as illustrated in Fig. 4 [24].

#### Training dataset

**Fig. 5:**
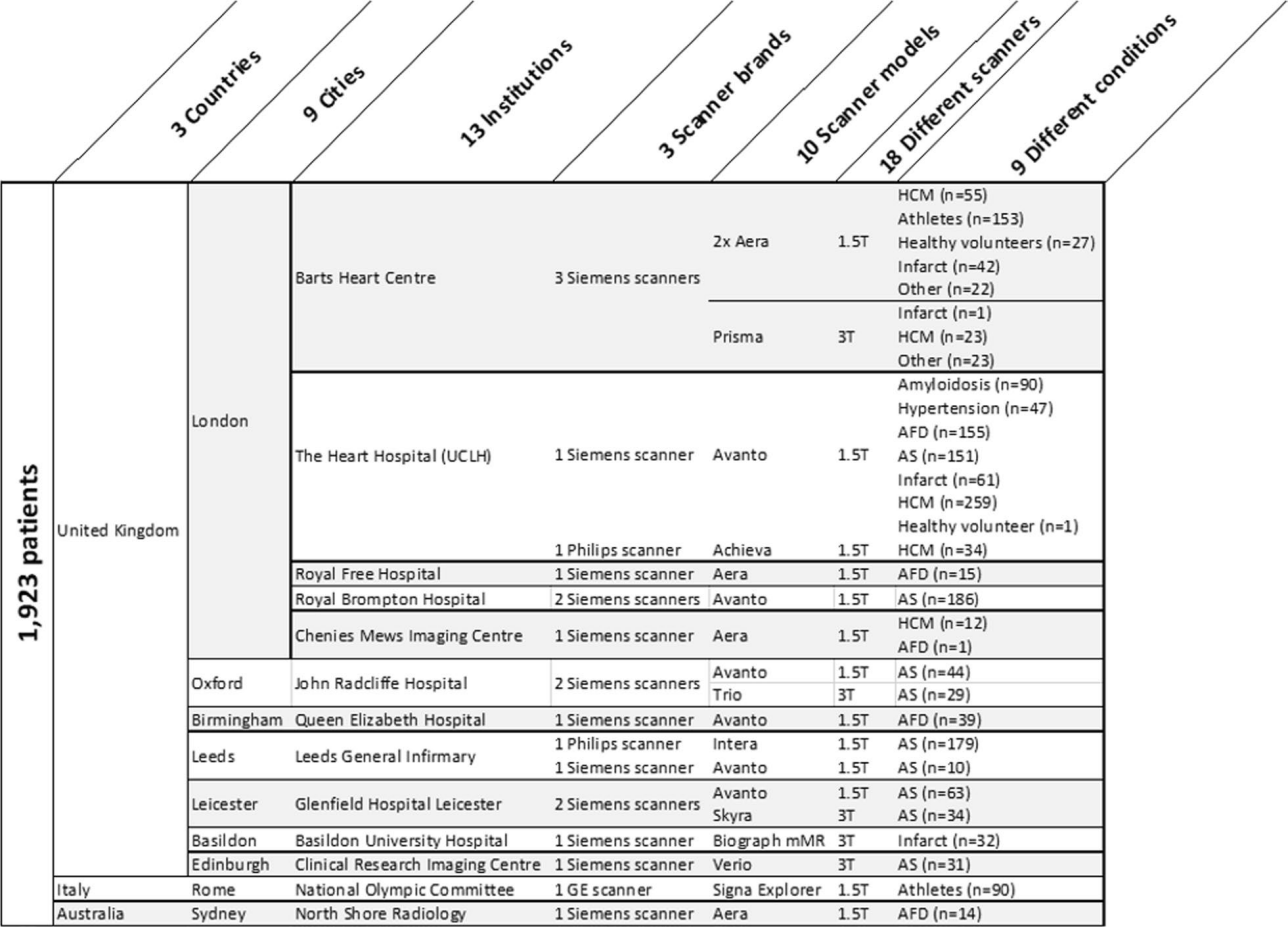
Composition of training data. List of countries, cities, institutions, scanner brand, scanner models and conditions (disease or healthy) used in the training dataset. *AFD* Anderson-Fabry Disease, *AS*  aortic stenosis, *HCM*  hypertrophic cardiomyopathy

We developed a generalizable scanner-agnostic and disease-independent model using anonymized data from imaging datasets of 1,923 patients previously recruited via written informed consent to clinical studies, all with approval from the local research ethics committees and complying to the principles of the Helsinki declaration. Subjects were scanned with two field strengths (1.5 and 3 T), three CMR manufacturers (Siemens Healthineers, Erlangen, Germany; Philips Healthcare, Best, the Netherlands; General Electric Healthcare, Chicago, Illinois, USA), 10 scanner models, 13 institutions from three countries (see Fig. 5). Cohorts were selected to balance health, physiological adaptation (athleticism), diseases with hypertrophy (aortic stenosis, hypertension, cardiac amyloidosis, Fabry disease, hypertrophic cardiomyopathy) and dilatation (myocardial infarction, dilated cardiomyopathy).

Images were segmented by three clinical cardiology trainees (CL, RH, YY) and reviewed and adjusted by a single expert with > 15 years’ clinical experience (JCM). Clinician segmentation was performed on cvi42 software (version 5.3.6, Circle Cardiovascular Imaging, Calgary, Alberta, Canada) using the semi-automated threshold tool with the ‘smoothed contour’ option enabled and post-hoc freehand correction as needed—the choice of tool was informed by previous work showing this to be most precise.[13] Segmentations followed standard convention where trabeculae and papillary muscles were considered part of the blood pool.

#### Model optimization by refining training segmentations

Following model parameter optimization, an iterative approach was adopted for model refinement. At each iteration of the model, expert readers (JBA, JCM, RhHD) used custom-built tools to inspect model segmentation predictions. We found that anatomically implausible segmentations could be traced back to the original training data and amending the training segmentation could remove such errors in subsequent models. The basal slice was particularly difficult and multiple approaches were trialed. For the final segmentation approach for training, we annotated the entire basal slice and one additional atrial slice, allowing the mitral annular plane correction algorithm to precisely define slice inclusion/exclusion with respect to the ventricle—a departure from conventional approaches [11].

#### LV metrics

The LV was segmented in each SAx image using volumes calculated from all SAx images in that cardiac phase. End-systole (the phase with smallest blood volume) and at end-diastole (largest volume) were identified and their volumes used to derive LV end diastolic volume (LVEDV), end systolic volume (LVESV), LVEF, and myocardial mass (LVM).

### Model evaluation

Machine learning model performance is often reported in terms of measurement accuracy (the closeness of a measure to its true value [25]) by measuring agreement (e.g. using the DICE coefficient) between model segmentations and those produced by a clinician(s). However, clinician segmentation is subjective and variable, making it unsuitable for use as a truth standard [26]. Since no other suitable truth standard exists, we concentrate on measurement precision (agreement of measures [25]), evaluated on a scan-rescan dataset. Since high measurement precision can be achieved with consistent but inaccurate segmentations, we also make a qualitative assessment of segmentation performance (as a surrogate for accuracy).

No images used in model evaluation had been included in the training data.

#### Measurement precision

Scan-rescan precision was measured using an independent dataset intended for benchmarking segmentations (we make these freely available at www.thevolumesresource.com) obtained on 109 subjects who were scanned, then removed from the scanner before being scanned again. The dataset contains multiple pathologies (32 myocardial infarctions, 17 LV hypertrophy, 17 cardiomyopathy, 8 cardio-oncology patients, 5 with chronic kidney disease, 30 healthy subjects), scanned at two field strengths at five institutions, as previously described [13].

A benchmark for human precision was obtained from segmentations performed by clinicians. First, all scan and rescan studies were combined into a single pool and presented in a randomized order to two trainees (YY, CL, 1–2 years CMR experience) and one expert (JCM, > 15 years CMR experience) who segmented each one using cvi42 software (version 5.3.8, Circle Cardiovascular Imaging)—using the semi-automated threshold tool with freehand correction and the mitral valve plane correction option enabled [13]. A further benchmark was obtained from the fully automated deep learning tool from a commercial software package (cvi42, version 5.11, Circle Cardiovascular Imaging).

#### Segmentation performance

Segmentation quality was assessed by two observers (JA, JCM) who identified any anatomically incorrect segmentations that affected ≥ 5% of overall cardiac volume. This allowed comparison of mis-segmentation rates between Machine, Human and cvi42.

#### Generalizability

In order to test the generalizability of the method to other patients, we also assessed the automated segmentations produced by our algorithm on an independent dataset of consecutive patients scanned as part of a clinical service at an independent center (University of Pittsburgh Medical Centre, Pittsburgh, Pennsylvania, USA) between June 2010 and March 2016 [27] Scans were excluded if non-standard cine imaging sequences were acquired, leaving a total of 1277 patients (55 ± 15 years, 58% males) with a range of (non-congenital) diseases—see Additional file 1: Table S2 for further details.

#### Pilot study for normal reference range calculation

A normative reference range depends on the method that was used to create it. As a pilot study, we calculated a sex- and age-stratified reference range for machine learning segmentations to serve as a benchmark for our patient cohorts. The values were obtained by applying the machine learning segmentation algorithm to CMR scans of 98 healthy subjects (no symptoms or history of cardiovascular disease or diabetes, normal electrocardiogram, blood pressure and CMR who underwent CMR (Avanto, 1.5 T, Siemens Healthineers).

### Statistical analysis

Statistical analysis was performed in R (version 3.5.3; R Foundation for Statistical Computing, Vienna, Austria). All continuous variables are expressed as means (with 95% confidence intervals in brackets) or medians (interquartile range) if the data is not normally distributed.

The concordance correlation coefficient (CCC) was used to assess agreement between human and machine segmentations for all LV metrics [28]. Intra-observer re-read variability (between pairs of identical scans) and scan-rescan variability (two separate scans of the same patient) were quantified using the within-subject coefficient of variation (CoV), calculated by the root mean squared method, and using n = 10,000 bootstrap samples to estimate standard errors and confidence intervals [29–31]. Mean absolute differences and Bland–Altman coefficients were also calculated for completeness. Sample size required to detect a clinical change was derived from the standardized difference for each LV metric with a power of β = 0.9 at a significance level of α = 0.05. The minimal detectable change between two scans was calculated by 1.96 x √2 × standard error of measurement (SEM).

## Results

### Performance

Human LV analysis took a median 13 min (interquartile range: 9–19 min) per study. Machine analysis took 20 s on a conventional workstation with NVIDIA GTX 1080Ti graphical processing unit.

**Fig. 6:**
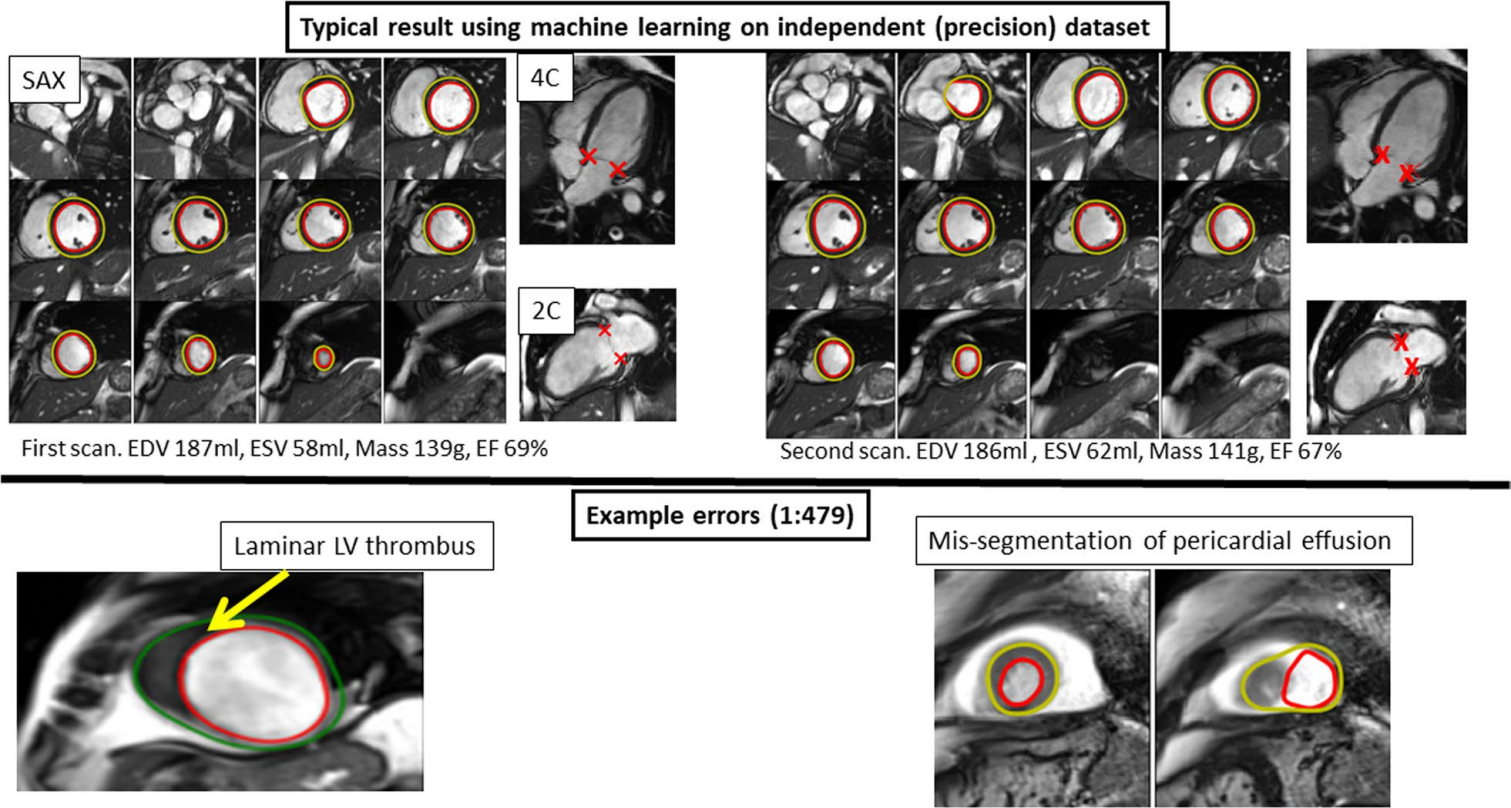
Example segmentations by machine learning algorithm. Top row: a pair of diastole images from the scan:rescan dataset that has been segmented by the automated algorithm. Note that the LV metrics are not exactly the same due to intrinsic variability in how slices are prescribed. Bottom left: example of an error (1 in 479 error rate) where laminar thrombus had been mis-identified as myocardium since this had not been ‘seen’ in the training data before. Bottom right: a mis-segmentation due to a pericardial effusion

Machine mis-segmentations were identified in the combined dataset of 72 of 34,486 (1 in 479) images [9 of 5058 in the Precision dataset and 63 of 29,428 in the Generalizability dataset]. Errors were seen in rare scenarios not encountered in training and in cases where contextual information assimilated by the human expert, was missed by the machine—examples include ventricular thrombus, artifact, effusions, poor image piloting, and unusual extracardiac findings (Fig. 6). There was also occasional over- or under-segmentation of the basal slice, caused by a change in breath-hold position between short- and long-axis acquisition.

cvi42 had a mis-segmentation rate of 89 in 5,058 (1 in 57) images on the Precision dataset, mostly because of missing contours, particularly at the base.

### Measurement bias

The LV metrics (LVEDV, LVESV, LVEF, LVM) derived from human and machine segmentations were highly correlated on the test (precision) dataset with a CCC range of 0.94 to 0.95. Absolute measurement differences between human and machine methods on the precision dataset were (LVEDV − 12 mls; LVESV − 3 mls; LVEF − 2%, LVM + 9 g)—see Additional file 1: Table S3. Because of the discrepancy, an algorithm-specific reference range was calculated as described below.

### Measurement precision

**Fig. 7:**
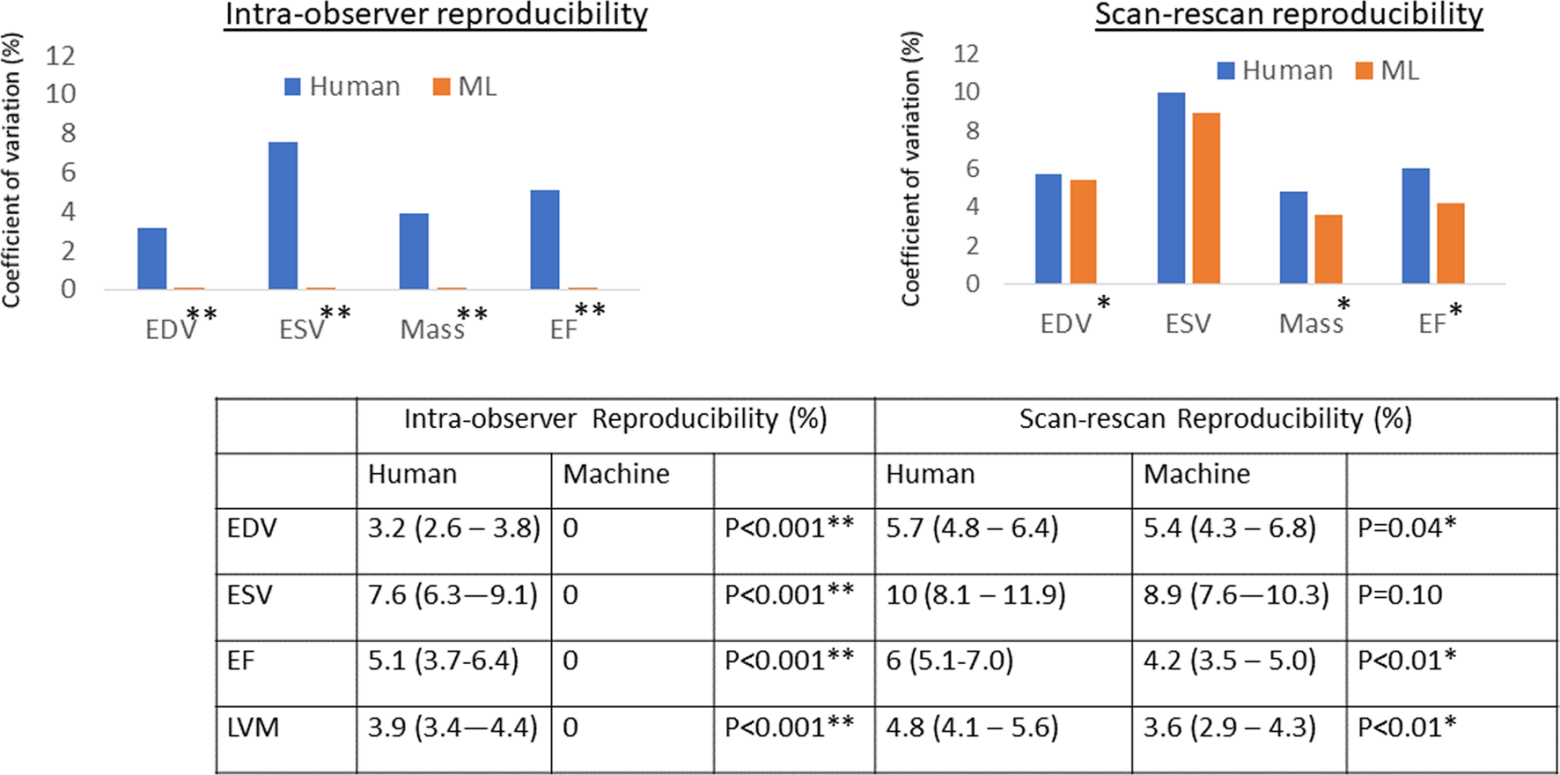
Machine and human precision evaluated on 109 subjects. Intra-observer reliability and scan-rescan repeatability, expressed as coefficient of variations (%) with 95% confidence intervals in brackets. Note that the intra-observer reproducibility is zero for all LV metrics. *Denotes statistical significance; ** denotes highly significant difference. *EDV* end diastolic volume, *ESV* end systolic volume, *EF* ejection fraction, *LVM* LV mass

Human intra-observer re-read variability (Fig. 7; Additional file 1: Table S4), had coefficient of variation (CoV) of LVEDV 3.2%, LVESV 7.6%, stroke volume 6.1%, LVEF 5.1%, LVM 3.9%. For machine, CoV were all 0% because the algorithm is deterministic. Scan-rescan CoV (Fig. 7) was significantly larger for human compared to machine across all parameters except for ESV, which was equivalent (for example LVEF 6.0% vs 4.2%, LVM 4.8% vs 3.6%; both *P* < 0.05). The increased machine precision translates to a better minimal detectable change (LVEF 8.7% to 7.0%; LVM 19.2 g to 13.7 g) and a potential reduction in clinical trial sample size by 46% if LVEF was used as an endpoint (see Additional file 1: Table S5). cvi42 software precision was significantly inferior to both human and machine for LV metrics (for example, LVEF 10.4%, LVM 8.8%; all p < 0.001 compared to both human and machine).

### Pilot study to define a reference range

Of the 98 healthy subjects, 52% were male, with a mean age of 50 ± 14 (range 20–76 years). The mean values of machine-derived LV metrics were: LVEF 65% (95% confidence interval 55–75%), LVEDV 145 ml, LVESV 51 ml, LVM 94 g. See Additional file 1: Table S**6** for age- and sex-stratified mean values and confidence intervals.

## Discussion

We present a fully automated method of CMR LV volumetric analysis and demonstrate that it has superior precision to a human expert. Widespread adoption has the potential to standardize global care, reduce the need for clinical expert time, and significantly reduce sample sizes for clinical trials.

Automated CMR analysis has already demonstrated faster performance with non-inferiority to humans [13]. Here, we demonstrate a generalizable algorithm with better-than-human precision with a substantial step-change that could impact both clinical and research work. Clinically, improved imaging biomarker precision builds confidence in quantitative analysis of cardiac structure and function and will help cascading clinical decisions that are based on cardiac measurement. For research, there is a potential reduction in required sample sizes, potentially accelerating therapeutic development. The automated method also permits retrospective analyses with considerable power—for example re-analyzing a 200 patient CMR study would take 60 min and could unearth findings previously masked by human analysis variability.

Machine errors were seen in circumstances not encountered in the training data, such as a laminar thrombus mimicking the LV wall, or a pericardial effusion resembling LV blood (Fig. 6; Additional file 1: Table S7). This in part represents a limitation when training data is collected from consented research subjects who, by definition, must be well enough to give consent. However, it also highlights that humans consider contextual information and use high-level executive function outside the scope of current AI systems. The method we present here has also has not been tested on patients with congenital heart disease and it is unlikely to generalize to such cases due to significant differences in structure and topology. This poses an interesting question about how to best model different diseases: do we create a separate model of each phenotype, or should they all be lumped together into a single model?

Sources of longitudinal variability in image-derived metrics can be grouped into three categories: variable image slice prescription by the radiographer, inconsistent analysis by the clinician, and physiological or pathological changes. Our goal is to minimize the first two sources (errors caused by inconsistency) so that we can focus on true physiological (or pathological) variability, which is crucial for serial assessment in clinical cardiology (e.g. monitoring for signs of cardiotoxicity during chemotherapy) and in clinical trials. Here, we have shown how image analysis variability can be minimized, but in future work we will extend this to automate the image acquisition process using deep learning to prescribe consistent image planes.

The difference between scan-rescan coefficients of variation (Fig. 7) and the mean absolute difference (Additional file 1: Table S4) may look small but these translate to a significant difference for both research studies and clinical care. As an example, if we used machine segmentations instead of human analysis in a clinical trial with an LVEF endpoint, we would need 46% fewer subjects to achieve the same statistical power. Minor differences in LV metric values also propagate in clinical care because normal values are treated as simple ‘cut-off’ values. Furthermore, the reported values represent the mean and larger differences are seen at an individual level.

Clinical adoption of machine learning methods is slow due to challenges with data access, integrating computer science and clinical domains, as well as validation, transparency, ethical and regulatory concerns [32]. Here, we have demonstrated how machine learning can be applied to an important medical problem, cardiac volumetric analysis by CMR, and its performance measured using a clinically important metric—precision. Unlike the majority of previous approaches, we directly compared machine performance to a clinician on the same independent data [19]. Such datasets could be a cornerstone of regulatory approval, where all new algorithms are systemically and independently evaluated and benchmarked against existing approaches.

There are significant differences between normative reference ranges for cardiac structure and function reported in the literature, (for example [33, 34]) and even small changes can lead to big differences in the number of subjects beyond cut-off thresholds. Much of this variability could be due to differences in sample populations (e.g. age, sex, comorbidities), imaging techniques (e.g. gradient echo vs. balanced steady state free procession), and measurement convention (papillary muscles considered as part of the LV blood pool vs. as myocardium). Experts also have their own biases, but so do automated algorithms which is why we believe that is important to report method-specific reference range. We performed a pilot study to estimate a normal reference range, but the number of patients, particularly in older age groups, is small so we aim to refine this in future using larger samples, such as UK Biobank.

### Limitations

Technical limitations of the algorithm include the method of co-registering the data from SAx, 2Ch and 4Ch images in 3D, which assumes consistent breath hold at the time of each acquisition, which may not always be the case. We will investigate ways of compensating for breath hold inconsistencies and integration into true 3D volumes in future work.

Other limitations include the limited number of human observers from a single centre used to benchmark the Precision dataset. This took 210 h of manual segmentation, but we acknowledge that validation with more clinicians from more centres is required before we can generalize the finding that the algorithm outperforms humans.

## Conclusion

We present an automated, generalized (any scanner and disease) method of measuring cardiac structure and systolic function that—without any intervention or correction—outperforms human analysis for precision.

## Supplementary Information

**Additional file 1: Table S1.** Technical details of each neural network model (U-net) used. **Table S2.** Demographics and descriptive analysis of the generalizability cohort with a total of 1,277 patients. BMI = body mass index; LVEDV = left ventricle end diastolic volume; LVEF = left ventricle ejection fraction; LVESV = left ventricle end systolic volume; LGE = late gadolinium enhancement; LVM = left ventricle mass. **Table S3.** Comparison of Mean values (standard deviation in brackets) for LV metrics computed using three different methods in the Precision dataset. LVEDV: left ventricular end-diastolic volume; LVESV: left ventricular end-systolic volume; LVEF: left ventricular ejection fraction; LVM: LVM: left ventricular mass; LVSV: left ventricular stroke volume. **Table S4.** Comparison of scan-rescan precision metrics between human, machine and cvi42. LVEDV: left ventricular end diastolic volume; LVESV: left ventricular end systolic volume; LVEF: left ventricular ejection fraction; LVM: left ventricular mass; LVSV: left ventricular troke Volume **Table S5.** Sample size calculation based on Precision dataset. sd = standardized difference. **Table S6.** Pilot Study for Normal reference range showing mean (95% confidence interval in bracket) for each LV metric for machine-derived CMR volumes from a set of 98 healthy subjects. The combined reference range is presented as well as sex- and age-stratified ranges. **Table S7.** Breakdown of segmentation error by type and location on the validation (precision) dataset, which contains a total of 5058 images.

## Data Availability

The datasets during and/or analyzed during the current study available from the corresponding author on reasonable request.

## Abbreviations

2Ch: Two-chamber
4Ch: Four-chamber
CCC: (Lin’s) Concordance correlation coefficient
CMR: Cardiovascular magnetic resonance
CNN: Convolutional neural networks
DICOM: Digital imaging and communications in medicine
LAx: Long axis
LVEDV: Left ventricular end-diastolic volume
LVESV: Left ventricular end-systolic volume
LVEF: Left ventricular ejection fraction
LVM: Left ventricular mass
MV: Mitral valve
SAx: Short axis
