# Supplementary material for: Precision measurement of cardiac structure and function in cardiovascular magnetic resonance using machine learning
Source: J Cardiovasc Magn Reson. 2022 Mar 10;24:16. doi: 10.1186/s12968-022-00846-4 (PMC8908603; doi:10.1186/s12968-022-00846-4)
Supplement: Supplementary file 1 — Additional file 1: Table S1. Technical details of each neural network model (U-net) used. Table S2. Demographics and descriptive analysis of the generalizability cohort with a total of 1,277 patients. BMI = body mass index; LVEDV = left ventricle end diastolic volume; LVEF = left ventricle ejection fraction; LVESV = left ventricle end systolic volume; LGE = late gadolinium enhancement; LVM = left ventricle mass. Table S3. Comparison of Mean values (standard deviation in brackets) for LV metrics computed using three different methods in the Precision dataset. LVEDV: left ventricular end-diastolic volume; LVESV: left ventricular end-systolic volume; LVEF: left ventricular ejection fraction; LVM: LVM: left ventricular mass; LVSV: left ventricular stroke volume. Table S4. Comparison of scan-rescan precision metrics between human, machine and cvi42. LVEDV: left ventricular end diastolic volume; LVESV: left ventricular end systolic volume; LVEF: left ventricular ejection fraction; LVM: left ventricular mass; LVSV: left ventricular troke Volume Table S5. Sample size calculation based on Precision dataset. sd = standardized difference. Table S6. Pilot Study for Normal reference range showing mean (95% confidence interval in bracket) for each LV metric for machine-derived CMR volumes from a set of 98 healthy subjects. The combined reference range is presented as well as sex- and age-stratified ranges. Table S7. Breakdown of segmentation error by type and location on the validation (precision) dataset, which contains a total of 5058 images. [file 12968_2022_846_MOESM1_ESM.docx]

**Additional Tables**

Table S1. Technical details of each neural network model (U-net) used

| **Model** | **Input image size** | **Output image type** | **Cost function used during training** | **Image Augmentation Parameters** |
| --- | --- | --- | --- | --- |
| **SAx diastole** | 192x192 pixels | Trinary image (0=blood, 1=myocardium, 2=background) | Categorical Cross-entropy | Scale = 1±0.1  Rotation = ±5^0^  Translation=±10 pixels |
| **SAx; all other phases** | 192x192 pixels | Binary image (0=blood, 1=background) | Categorical Cross-entropy | Scale = 1±0.1  Rotation = ±5^0^  Translation=±10 pixels |
| **2 chamber** | 256x256 pixels | Two-channel Gaussian-weighted distance maps (each channel represents one mitral annular point) | Root mean square | Scale = 1±0.05  Rotation = ±10^0^  Translation=±15 pixels |
| **4 chamber** | 256x256 pixels | Two-channel Gaussian-weighted distance maps | Root mean square | Scale = 1±0.05  Rotation = ±10^0^  Translation=±15 pixels |

Sax, short axis.

Table S2. Demographics and descriptive analysis of the generalizability cohort with a total of 1,277 patients. BMI=body mass index; LVEDV=left ventricle end diastolic volume; LVEF=left ventricle ejection fraction; LVESV=left ventricle end systolic volume; LGE=late gadolinium enhancement; LVM=left ventricle mass;.

| Age (Median, IQR), years | 57 [45-66] |
| --- | --- |
| Male (%) | 58% |
| BMI median, [IQR] (kg/m2) | 28.7 [24.9-34.0] |
| Heart failure | 31% |
| Hypertension | 51% |
| Diabetes | 22% |
| LVEDV median, [IQR] (ml) | 166 [132-211] |
| LVESV median, [IQR] (ml) | 69 [47-113] |
| LVEF median, [IQR] (%) | 57 [44-66] |
| LVM median, [IQR] (g) | 129 [99-162] |
| Infarct pattern on LGE | 21% |
| Non-infarct pattern on LGE | 22% |

*Table S3. Comparison of Mean values (standard deviation in brackets) for LV metrics computed using three different methods in the Precision dataset. LV*EDV: left ventricular end-diastolic volume; LVESV: left ventricular end-systolic volume; LVEF: left ventricular ejection fraction; LVM: LVM: left ventricular mass; LVSV: left ventricular stroke volume.

|  | LVEDV, ml | LVESV, ml | LVSV, ml | LVEF, % | LVM, g |
| --- | --- | --- | --- | --- | --- |
| Human | 158 (49) | 67 (41) | 90 (23) | 59 (12) | 142 (43) |
| Machine | 170 (51) | 70 (43) | 100 (25) | 61 (12) | 133 (40) |
| cvi42 | 178 (54) | 82 (48) | 96 (26) | 56 (12) | 116 (33) |

Table S4. Comparison of scan-rescan precision metrics between human, machine and cvi42. LVEDV: left ventricular end diastolic volume; LVESV: left ventricular end systolic volume; LVEF: left ventricular ejection fraction; LVM: left ventricular mass; LVSV: left ventricular troke Volume.

| **Coefficient of variation, % (95% CI)** | | | | | |
| --- | --- | --- | --- | --- | --- |
|  | LVEDV | LVESV | LVSV | LVEF | LVM |
| Human | 5.7 (4.8, 6.8) | 10 (8.1,11.9) | 9.4 (7.8,11.0) | 6 (5.1, 7.0) | 4.8 (4.1, 5.6) |
| Machine | 5.4 (4.3, 6.4) | 8.9 (7.6, 10.3) | 7 (5.8, 8.2) | 4.2 (3.5, 5.0) | 3.6 (2.9, 4.3) |
| cvi42 | 11.4 (6.5, 15.6) | 18.4 (11.3, 25.3) | 15.2 (11.7, 18.6) | 10.4 (6.8, 14.0) | 8.8 (6.3, 11.2) |
|  |  |  |  |  |  |
|  | | | | | |
| **Mean absolute difference (std dev)** | | | | | |
|  | LVEDV, ml | LVESV, ml | LVSV, ml | LVEF, % | LVM, g |
| Human | 9 (8) | 6 (6) | 9 (8) | 4 (3) | 7 (7) |
| Machine | 8 (8) | 5 (5) | 7 (7) | 3 (2) | 5 (5) |
| CVI42 | 16 (20) | 11 (14) | 14 (15) | 5 (5) | 9 (10) |
|  |  |  |  |  |  |
|  |  |  |  |  |  |
| **Bland Altman limits of agreement** | | | | | |
|  | LVEDV | LVESV | LVSV | LVEF | LVM |
| Human | -21.4, 25.5 | -17.2, 16.8 | -20.2, 25 | -8.7, 9.9 | -19.1, 18.6 |
| Machine | -21.5, 24.5 | -14.5, 14.3 | -17.3, 20.6 | -6.7, 7.3 | -13.9, 13.1 |
| CVI42 | 3.4, -46.8 | -0.7, -35 | 4, -36 | 0.6, -13.9 | -0.1, -27 |

Table S5. Sample size calculation based on Precision dataset. sd=standardized difference.

|  | LVEDV | |  | LVESV | |  | LVSV | |  | LVEF | |  | LVM | |
| --- | --- | --- | --- | --- | --- | --- | --- | --- | --- | --- | --- | --- | --- | --- |
|  | 5ml change | |  | 5ml change | |  | 5ml change | |  | 3% change | |  | 5g change | |
|  | Sd | N |  | sd | n |  | sd | n |  | Sd | n |  | sd | n |
| Expert | 12 | 62 |  | 9 | 34 |  | 12 | 58 |  | 5 | 61 |  | 10 | 41 |
| Neural Network | 12 | 60 |  | 7 | 25 |  | 10 | 41 |  | 4 | 33 |  | 7 | 22 |

Table S6. Pilot Study for Normal reference range showing mean (95% confidence interval in bracket) for each LV metric for machine-derived CMR volumes from a set of 98 healthy subjects. The combined reference range is presented as well as sex- and age-stratified ranges.

| **Combined Reference Range** | |
| --- | --- |
| LVEDV, ml | 145 (82-209) |
| LVESV, ml | 51 (22-81) |
| LVSV, ml | 94 (54-134) |
| LVM, g | 94 (45-154) |
| LVEF, % | 65 (55-75) |

|  | **Males** | | | | |  |
| --- | --- | --- | --- | --- | --- | --- |
| **Males** | 20-29 years | 30-39 years | 40-49 years | 50-59 years | 60-69 years | 70-79 years |
| **Total n=48** | n=4 | n=6 | n=15 | n=11 | n=9 | n=3 |
|  | **Absolute values** | | | | | |
| LVEDV, mL | 184  (134-234) | 176  (120-233) | 169  (105-232) | 161  (91-231) | 154  (77-230) | 146  (63-229) |
| LVESV, mL | 70  (45-95) | 66  (37-94) | 61  (30-93) | 57  (23-92) | 53  (15-91) | 49  (8-90) |
| LVSV, mL | 114  (82-146) | 111  (74-147) | 107  (67-148) | 104  (59-148) | 100  (52-149) | 97  (44-150) |
| LVEF, % | 62  (54-70) | 63  (54-72) | 64  (53-74) | 65  (53-76) | 66  (53-78) | 66  (53-80) |
| LVM, g | 114  (82-145) | 114  (78-149) | 114  (74-154) | 114  (70-158) | 114  (65-162) | 114  (61-166) |
|  | **Indexed to body surface area** | | | | | |
| LVEDVI, mL/m^2^ | 90  (71-110) | 87  (65-109) | 83  (59-108) | 80  (53-107) | 76  (47-106) | 73  (41-105) |
| LVESVI, mL/m^2^ | 34  (23-45) | 32  (20-44) | 30  (17-44) | 28  (13-43) | 26  (10-43) | 25  (7-42) |
| LVSVI, mL/m^2^ | 56  (44-69) | 55  (40-69) | 53  (37-69) | 51  (34-69) | 50  (31-69) | 48  (27-69) |
| LVMI, g/m^2^ | 56  (45-66) | 56  (44-68) | 56  (43-69) | 56  (42-71) | 56  (40-73) | 57  (39-74) |

|  | **Female** | | | | |  |
| --- | --- | --- | --- | --- | --- | --- |
| **Females** | 20-29 years | 30-39 years | 40-49 years | 50-59 years | 60-69 years | 70-79 years |
| **Total n=45** | n=6 | n=8 | n=9 | n=10 | n=7 | n=5 |
|  | **Absolute values** | | | | | |
| LVEDV, mL | 136  (106-166) | 131  (97-165) | 126  (88-164) | 121  (80-163) | 116  (71-162) | 11  (62-161) |
| LVESV, mL | 48  (33-63) | 46  (29-63) | 44  (25-64) | 43  (21-64) | 41  (18-64) | 39  (14-64) |
| LVSV, mL | 88  (69-107) | 85  (64-107) | 82  (58-106) | 79  (52-105) | 76  (47-105) | 73  (41-104) |
| LVEF, % | 65  (58-72) | 65  (57-73) | 65  (56-74) | 65  (55-75) | 65  (55-76) | 65  (54-77) |
| LVM, g | 77  (57-96) | 76  (53-98) | 75  (50-100) | 74  (47-102) | 73  (43-103) | 72  (40-105) |
|  | **Indexed to body surface area** | | | | | |
| LVEDVI, mL/m^2^ | 81  (68-94) | 78  (63-93) | 75  (58-92) | 72  (54-91) | 69  (49-90) | 66  (44-88) |
| LVESVI, mL/m^2^ | 28  (21-36) | 27  (19-36) | 26  (17-36) | 25  (15-36) | 24  (13-36) | 23  (11-36) |
| LVSVI, mL/m^2^ | 53  (44-62) | 51  (41-61) | 49  (38-60) | 47  (35-59) | 45  (32-59) | 43  (28-58) |
| LVMI, g/m^2^ | 46  (37-54) | 45  (35-55) | 45  (33-56) | 44  (32-57) | 44  (30-57) | 43  (28-58) |

Table S7. Breakdown of segmentation error by type and location on the validation (precision) dataset, which contains a total of 5058 images.

|  | *Missing contour* | | |  | *Poor quality contour* | | |  | *Wrong structure segmented* | | |
| --- | --- | --- | --- | --- | --- | --- | --- | --- | --- | --- | --- |
|  | *Basal* | *Mid* | *Apical* |  | *Basal* | *Mid* | *Apical* |  | *Basal* | *Mid* | *Apical* |
| *Machine* | *3* | *1* | *0* |  | *0* | *1* | *1* |  | *2* | *0* | *1* |
| *cvi42* | *51* | *10* | *12* |  | *6* | *4* | *6* |  | *0* | *0* | *0* |
